# Effectiveness of ballast water management systems in the Great Lakes based on a paired uptake-discharge sample design

**DOI:** 10.1007/s10661-025-14032-3

**Published:** 2025-05-02

**Authors:** Oscar Casas-Monroy, Jiban C. Deb, Jocelyn Kydd, Robin Rozon, Sean Yardley, Sophie Crevecoeur, Sarah A. Brown, John A. Darling, Sarah A. Bailey

**Affiliations:** 1 Great Lakes Laboratory for Fisheries and Aquatic Sciences, Fisheries and Oceans Canada, 867 Lakeshore Road, Burlington, ON L7S 1A1 Canada; 2 Northern Hardwoods Research Institute Inc, 165 Boulevard Hébert, Edmundston, NB E3V 2S8 Canada; 3 Watershed Hydrology and Ecology Research Division, Environment and Climate Change Canada, 867 Lakeshore Road, Burlington, ON L7S 1A1 Canada; 4 Oak Ridge Institute for Science and Education, P.O. Box 117, Oak Ridge, TN 37831 USA; 5 Center for Environmental Measurement and Modeling, US EPA Office of Research and Development, 109 T.W. Alexander Dr, Research Triangle Park, Durham, NC 27709 USA

**Keywords:** Ballast water management, Ballast water treatment, Freshwater ecosystems, Plankton, Treatment efficacy

## Abstract

Ships’ ballast water is a major vector for aquatic non-native species, particularly in the Great Lakes and St. Lawrence River (GLSLR) region. Effective September 8, 2024, international ships must comply with an international convention limiting live organisms in discharged ballast water, typically by installing onboard ballast water management systems (BWMS). This study assessed BWMS effectiveness in the GLSLR using a paired uptake-discharge sample design (before and after treatment), to evaluate live organism abundance in the two regulated size classes, and plankton diversity, using microscopy and high-throughput sequencing. Samples from 10 ships (11 paired events) collected in 2019 and 2022 revealed uptake abundances of living organisms up to 107,600 organisms/m^3^ for the large (≥ 50 μm) and 169 organisms/mL for the small (≥ 10 to < 50 µm) size classes, respectively. While all discharge samples met the small size class limit, four samples exceeded the large size class limit, with BWMS reducing abundances by at least 98%. High uptake abundance and BWMS filter mesh size were key predictors of organism abundance in discharge samples, highlighting potential challenges in meeting the discharge standard in eutrophic waters. BWMS also reduced plankton diversity in discharge samples compared to uptake samples, with molecular methods showing greater sensitivity than microscopy but potentially detecting DNA from both live and dead organisms. The findings underline the need for further research to address BWMS performance in different water qualities and to improve technology. Complementary use of microscopy and molecular methods offers a comprehensive approach to evaluating plankton diversity and BWMS effectiveness.

**Supplementary Information:**

The online version contains supplementary material available at 10.1007/s10661-025-14032-3.

## Introduction

Global maritime trade has facilitated the movement of goods across international waters, stimulating economic growth and interconnectivity (Hoffmann et al., 2017). However, the increase in maritime activity has also raised significant environmental concerns regarding the potential introduction of non-native species and pathogens to new ecosystems due to the transfer of ballast water (Seebens et al., 2013). Ballast water is essential for maintaining ship stability, trim, and structural integrity during voyages, but it can unintentionally transport harmful aquatic organisms and pathogens from one region to another (Bailey, 2015). Particularly for the North American Great Lakes, such introductions of non-native species pose severe threats to biodiversity, ecosystem function, and various economic sectors including fisheries, aquaculture, and tourism (Colautti et al., 2014; Ricciardi & Macisaac, 2000).

To address this issue, the International Maritime Organization (IMO) adopted the International Convention for the Control and Management of Ships’ Ballast Water and Sediments in 2004 (International Maritime Organization, 2004). As of September 8, 2024, ships on international voyages must comply with Regulation D-2, which limits the number of viable organisms in discharged ballast water. Regulation D-2 states that ships must contain < 10 viable organisms/m^3^ ≥ 50 μm in minimum dimension (typically zooplankton) and < 10 viable organisms/mL ≥ 10 μm and < 50 μm in minimum dimension (typically phytoplankton) (International Maritime Organization, 2004). Most ships have installed and operate a ballast water management system (BWMS) to achieve compliance.

Previous research has primarily focused on evaluating compliance of ballast water discharges following the use of BWMS (Bailey et al., 2022; Casas-Monroy & Bailey, 2021; Drillet et al., 2023). Although compliance with the D-2 limits is verified during type-approval and commissioning testing of BWMS, non-compliance persists under real-world conditions encountered by ships in operational service (Bailey et al., 2022; Drillet et al., 2023; Outinen et al., 2024). Factors such as environmental conditions, water quality, and incorrect installation, operation, or maintenance of BWMS may influence treatment efficacy. To fully understand BWMS performance, discharge samples (after treatment) must be assessed in comparison to uptake water (before treatment). Uptake samples provide a baseline, while discharge samples reveal the effect of treatment on ballast water prior to release into the environment. Examining paired uptake and discharge samples allows for better investigation of the cause of non-compliant samples, facilitating development of strategies to improve ballast water management and minimize the introduction of harmful species to aquatic ecosystems.

Ballast water operations in the GLSLR region have played a significant role in spreading aquatic non-native species (Chan et al., 2014; Kvistad et al., 2019). The extensive network of ports in the GLSLR facilitates the rapid dispersal of species, allowing them to travel long distances they would otherwise be unlikely to reach naturally (Bradie et al., 2021; Kvistad et al., 2019; Rup et al., 2010). While utilizing BWMS can lower invasion risk, meeting regulatory standards is an ongoing challenge and additional studies are needed to fully characterize the relationship between ballast water management practices and invasion risk across different taxonomic groups (Bradie et al., 2021; DFO, 2020).

To address these challenges, past research has typically relied on traditional methods such as microscopy to identify non-native species in ballast water (Egan et al., 2015). However, advancements in molecular diagnostic tools, such as high-throughput sequencing, have emerged as complementary methods for assessing community composition and biodiversity by amplifying and sequencing short, standardized DNA fragments directly from environmental samples (Zaiko et al., 2018). The application of molecular analysis in invasion ecology is still a relatively new field, but DNA metabarcoding can provide more comprehensive species inventories than traditional morphology-based methods, by capturing taxa that are small, damaged, or difficult to identify (James et al., 2024) if sufficient reference DNA libraries are available (Briski et al., 2016).

This study aimed to evaluate the effectiveness of BWMS at reducing the concentration and diversity of organisms in discharged ballast water on operational ships transiting the GLSLR under real-world conditions during 2019 and 2022. Alongside traditional microscopy, high-throughput sequencing of the cytochrome c oxidase I (COI) and 18S rRNA genes was employed as a supplement to improve the identification of zooplankton and phytoplankton organisms in ballast water respectively, focusing on taxonomic groups (spanning various taxonomic levels, from phyla to genera, as shown in Online Resource) that may persist in ballast water after treatment. Analyzing organism diversity before and after ballast water treatment reveals community shifts and taxonomic biases beyond D-2 compliance. While the D-2 limits focus on size and viability, species-level data may provide a better understanding of ecological impacts and treatment effectiveness. Monitoring diversity helps identify resilient organisms that may survive treatment and persist in the environment, potentially posing biosecurity risks upon discharge (Ardura et al., 2021). This research contributes to ongoing efforts to understand the effectiveness of ballast water management strategies, implement measures to further improve BWMS performance and reliability, and enhance environmental protection of the GLSLR ecosystem.

## Materials and methods

### Sample collection

**Fig. 1:**
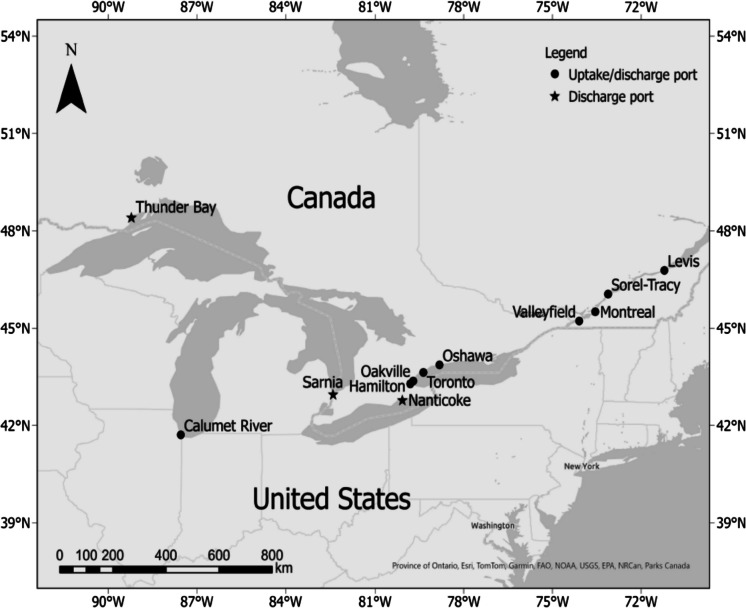
Ballast water sampling locations in the Great Lakes and St. Lawrence River region. Twelve ports served as either source or discharge sites for ballast water sample collection from ships arriving during May–November 2019 and 2022

Ballast water samples were opportunistically collected from ships arriving at 12 GLSLR ports during two sampling periods (May–November, 2019 and 2022) (Fig. 1). Ballast water sampling was conducted using designated sampling port(s) situated in the engine room, pump room, or on deck. During ballast uptake, 11 samples of untreated, incoming harbor water were obtained, consisting of at least 1 m^3^ volume. During ballast discharge (at least 24 h later), 11 samples were collected from the sampling port located near the overboard discharge point (treated ballast water from the same tank that was being filled at the time of uptake sampling; up to 3 m^3^ volume).

For organisms ≥ 50 μm (large size class), an in-line large volume collection device was used to collect ballast water samples following the protocol established by Bailey et al. (Bailey et al., 2022). Briefly, each 1-m^3^ sample was concentrated using a 35-μm mesh plankton net measuring 30 cm in diameter, submerged within a 75-L plastic container filled with ambient ballast water. The container has two drain lines, allowing processed water to be directed to a disposal location designated by the ship’s crew (e.g., ship's bilge). To minimize flow turbulence and potential harm to organisms, sampling occurred at sub-isokinetic flow rates, meaning the sample flow velocity was less than the main ballast pipe’s velocity (Wier et al., 2015). Once 1000 L of water was filtered, the outside of the plankton net was rinsed with 10-μm filtered ballast water to collect the sample into the cod end, which was then transferred to a 1-L plastic bottle. For discharge samples, this process was repeated up to three times, depending on available ballast discharge volume. Each sampling event was conducted for a 45–60-min continuous period during regular ballast operations to enhance representativeness, considering potential in-tank stratification and plankton patchiness (Bailey & Rajakaruna, 2017).

The large volume collection device also employed a small sample probe to divert a subsample of water to a small sample collection device (Moser et al., 2018) for concurrent collection of at least 16.1 L of unconcentrated water throughout the sampling period. This unconcentrated water volume was well-mixed, a 1-L subsample was extracted to estimate the abundance of live organisms ≥ 10 to < 50 μm (small size class), a 10-L subsample was concentrated and preserved for subsequent taxonomic enumeration using microscopy techniques, 5 L was allocated for molecular analysis, and a 50-mL subsample was utilized for measurements of various water quality parameters, including ultraviolet transmittance (UVT), total residual oxidant (TRO), and salinity.

Throughout the sample collection period, a YSI EXO multi-parameter water quality sonde (YSI Incorporated, Yellow Springs, OH) was immersed in the 75-L container, continuously monitoring various water quality parameters. Following collection, samples were wrapped in thermal blankets and stored in dark, insulated coolers to maintain ambient water temperature and minimize organism mortality. Ballast water samples were transported off the ship for immediate analysis (within 6 h of sample collection). Once at the lab, the concentrated net samples were divided for microscopy and molecular analysis of the large size class. Each sample was mixed by gentle inversion (five times) prior to splitting; two-thirds of the sample volume were allocated for live microscopy counts and one-third was preserved in 95% ethanol (ethyl alcohol, Greenfield Global Inc.) for later molecular analysis. For the small size class, the 10-L ballast water sample was concentrated using a 10-μm mesh sieve, poured into a 250-mL brown plastic bottle and preserved with 3.75 mL of Lugol solution (Sigma–Aldrich Corporation) for later taxonomic analysis based on morphology.

### Sample analysis—live counts

For the large size class, the initial step of microscopy counts involved examining a 1-mL aliquot of the sample to establish the approximate organism concentration. Each sample was further concentrated (using a 35-μm mesh) or diluted (using 10-μm filtered ballast water), as required, to achieve a target density of 25–50 organisms/mL for optimal microscopy analysis. Multiple aliquots (0.5 to 2.5 mL) were then transferred to individual channels of a modified Bogorov counting chamber (Hydro-Bios Apparatebau GmbH, Germany) using an Eppendorf pipette equipped with a wide-bore tip. A 10-µL aliquot of 50-μm dyed green aqueous fluorescent microspheres (Fluoro-Max; ThermoScientific, Waltham, USA), suspended in double-distilled water at 100 mg/mL, was added to each channel as a size reference. Each channel was then examined under a Nikon SMZ800N Zoom Stereomicroscope at magnifications ranging from 30x to 80x. For uptake samples, the number of live individuals was calculated by subtracting the number of dead organisms from a total count. Conversely, only live organisms (individuals exhibiting movement or responding to stimuli - poking organisms with an insect pin mounted on a handle) (NSF International, 2010) were enumerated for discharge samples. The sample volume assessed ranged from 700 to 1999 L. Cumulative counts were subsequently converted to abundance per cubic meter based on the initial sample volume, applied concentration or dilution factor, and the fraction of the sample analyzed. For discharge samples, the abundance of live organisms was calculated cumulatively across the (up to) three replicates.

For the small size class, both uptake and discharge samples were examined by epifluorescence microscopy using fluorescein diacetate (FDA, Sigma-Aldrich Canada, Oakville, Ontario) as a vital marker (Adams et al., 2014). An FDA primary solution was made by combining 50 mg of solid powder FDA with 10 mL of reagent grade dimethyl-sulfoxide (DMSO; Sigma-Aldrich Canada, Oakville, Ontario), for a final concentration of 12.0 mM. Further, an FDA working solution was made through the addition of 10.0 μL of the FDA primary solution to 1.0 mL of distilled water, for a final working solution concentration of 120 μM. A 5-mL aliquot was removed from a well-mixed 1-L sample of unconcentrated ballast water, and 417 μL of working solution was added to the aliquot. The aliquot was incubated in the dark for 10 min; following incubation, 1 mL of the aliquot was transferred to a Sedgewick-Rafter counting chamber (Wildlife Supply Company, Yulee, Florida, USA). A 1-µL aliquot of 10 μm and 50 μm dyed green aqueous fluorescent microspheres (Fluoro-Max; ThermoScientific, Waltham, USA), suspended in double-distilled water at 100 mg/mL, was added to the counting chamber as a size reference. Six 1-mL aliquots were examined using a Zeiss Axio Vert.A1 microscope (Carl Zeiss Canada, Ltd, Toronto, Ontario, Canada), equipped with a LED Module (470 nm) and filter for green fluorescent protein (filter set 38, excitation 495 nm, emission 517 nm). All fluorescing organisms were counted in the entire chamber within 20 min. The mean abundance of live small size class organisms was calculated from the six 1-mL aliquots.

### Diversity

#### Microscopy

For the large size class, taxonomic identification based on morphology was limited to a coarse assessment using low-magnification stereoscope microscopy during live counts (e.g., copepods, nauplii, rotifers, etc.), and the number of individuals observed within those broad groups was recorded (as live or dead).

For the small size class, only preserved samples were processed to examine community composition using the Utermöhl method (Utermöhl, 1958). Intact cells bigger than 10 μm with clearly visible cell content were counted as “potentially viable” organisms at the time of preservation. Samples were observed using a Zeiss Axio Vert.A1 inverted transmitted light/reflected light fluorescence microscope (with fluorescence excitation based on LED modules; Carl Zeiss Canada Ltd., Toronto, ON, Canada) at 250x to 1000x magnification. Taxonomic identification follows Carty (Bellinger, 2015; Carty, 2014).

#### Metabarcoding

For the large size class, 1/3 of ballast water samples (split before live counts) were processed for high-throughput sequencing metabarcoding of the COI gene. Briefly, samples were filtered, and DNA was extracted using a DNeasy Power Soil Pro kit (Qiagen N.V., USA) following standard kit protocol. A fragment of the COI gene was amplified using the universal reverse primer jgHCO2198 (Geller et al., 2013) and a modified version of the mlCOIintF forward primer (Leray et al., 2013) (Jon Geller, personal communication). Additionally, heterogeneity spacers were added to the primers to allow for increased sequencing diversity. Amplicons were cleaned, dual-indexed, and prepared for paired-end 2 × 300 Illumina MiSeq (Illumina Inc., USA) sequencing in house. Raw Illumina sequences were imported into QIIME2 (version 2023.7 (Bolyen et al., 2019)) and trimmed, denoised, and dereplicated using DADA2 (Callahan et al., 2016). Taxonomy was assigned to amplicon sequence variants (ASVs) using the QIIME2 classify-consensus-blast BLAST + classifier (Camacho et al., 2009) and the COInr database (Meglécz, 2023), which contains combined BOLD and NCBI reference sequences. The mkCOInr tool (Meglécz, 2023) was used to customize the COInr database to contain only sequences that covered the COI region amplified by the study primers. Only taxonomic assignments with a percent identity > 80% and a query coverage > 95% were included in the final dataset. Non-metazoan taxa (e.g., Fungi, Algae) were removed to facilitate comparison with morphological analysis.

For the small size class, a total of 5 L of uptake or discharge ballast water from each ship was used for genomic (DNA metabarcoding) analysis during 2022 only. Whole ballast water samples were filtered in triplicate onto polycarbonate filters (0.2 μm pore size, 47 mm diameter, Fisher Scientific, Canada) and preserved until DNA extraction. Filters were dried at 35 °C for 15 min and stored in foil envelopes in a plastic container with silica gel (de Vargas et al., 2020), and DNA was extracted using a DNeasy Power Kit (Qiagen N.V., USA) following standard kit protocol. The V4 region of the 18S rRNA gene was amplified with primers E572 F (CYGCGGTAATTCCAGCTC) and E1009R (AYGGTATCTRATCRTCTTYG) and sequenced on an Illumina MiSeq platform (Illumina Inc., USA) following a pair-end approach at the Integrated Microbiome Resource (IMR) located at Dalhousie University in Halifax, Nova Scotia. The 18S rRNA gene sequences were analyzed following the DADA2 pipeline (Callahan et al., 2016) using R (R Core Team, 2023) (Team, 2023) on the high-performance computing environment of Shared Services Canada in Dorval, Quebec. Non-biological sequences of the primers were removed with cutadapt (Martin, 2012). For each sequencing sample, raw read quality profiles were assessed and all low-quality bases at the end of the reads were trimmed at 200 bp with a truncQ score of 11. Sequences with a maximum expected error (maxEE) > 2 were removed, and high-quality sequences were merged into an amplicon sequence variant (ASV) using the mergePairs command with the option justConcatenate = TRUE since the amplicon was too short for an overlap. Chimeras were removed with the “removeBimeraDenovo” command, and taxonomy was assigned using the SILVA v128 18S database to select eukaryotic phytoplankton (identity ≥ 90%). Finally, an ASV table was constructed merging with metadata such as event ID and ballast water operations (uptake/discharge) (Online Resource 3).

### Statistical analysis

Generalized linear models employing both Quasi-Poisson and negative binomial distributions, which are recommended for overdispersed ecological count data (Hoef et al., 2003; Warton et al., 2016), were compared to select the most suitable model to investigate the relationship between the abundance of organisms in ballast water discharge samples and factors potentially influencing BWMS performance, for each size class. To inform the model selection process, several criteria were evaluated, including the significance of independent variables, residual deviance, basic diagnostic plots, and the Akaike information criterion; additionally, homoscedasticity was examined. It was hypothesized that the independent variables in the selected model such as BWMS type, BWMS filter size, ballast age (in days), BWMS age (in years), disinfection methods, sampling duration, EXO turbidity, EXO fDOM, ultraviolet transmittance (UVT), and abundance of organisms in uptake samples do not significantly contribute to explaining the variability in the response variable (null hypothesis).

Diversity was assessed using the Shannon diversity index (H′), calculated for each sample using taxonomic results from microscopy and molecular analysis. This index accounts for both species richness and evenness within the community. To examine whether diversity was significantly different between uptake and discharge samples, as well as between laboratory techniques, two-sample Welch’s *t* tests were performed. This variant of the *t* test was chosen as it does not assume equal variances between groups with unequal sample sizes.

## Results and discussion

A total of 22 ballast water samples (11 pairs of uptake/untreated and discharge/treated samples) were collected from 10 ships transiting the GLSLR region in 2019 and 2022 (see Table 1). Among the sampled ships, four were tankers—three with coastal routes and one exclusively transiting within the GLSLR. The remaining six ships were bulk carriers all with international transoceanic routes. All of the BWMSs used filtration prior to disinfection, with nominal mesh sizes ranging from 20 to 50 μm (Table 1). Nine ships had BWMS that applied physical treatment (ultraviolet radiation), while one ship had a BWMS that used chemical injection (chlorination). Three tests were performed with Optimarin systems, five tests were performed with Alfa Lava systems (four with Pureballast 3.1 and one with Pure ballast 3.3), one test with a BioUv (UV BioSea) system, and one with a JFE (BallastAce) system. The BWMS using chlorine for disinfection was equipped with a neutralization system to manage residual chlorine in ballast water prior to discharge. All ballast water discharge samples met the maximum allowable TRO level of less than 0.1 mg/L, as specified during the IMO Type Approval process.

**Table 1:** Summary of the ballast water histories and sampling outcomes, including ballast water management system (BWMS) details, for each test conducted during 2019 and 2022. All BWMS applied filtration + ultraviolet irradiation (UV) except one (which used filtration + chlorine injection). T. O. V. = total overboard volume discharged during the time of sample collection. Rated capacity is the maximum flow rate specified for the BWMS model, while the actual flow rate was the average flow rate during sampling

Sampling year	Event ID	Uptake location	T.O.V (m^3^)	BWMS Mesh size (µm)	BW age (days)	Rated capacity (m^3^/h)	Actual flow rate (m^3^/h)	Uptake Large size org./m^3^	Discharge Large size org./m^3^	Reduction Large size Class (%)	Uptake Small size org./mL	Discharge Small size org./mL	Reduction Small size Class (%)
2019	1	Hamilton, ON	765	40	5	1380	957	2168	12	99.5	2.5	0.0	100
2019	2	Calumet River, IL	792	40	5	1380	990	101,039	293	99.7	29.4	0.5	98.2
2019	3	Valleyfield, QC	418	20	2	600	440	4762	3	99.9	6.0	0.0	100
2019	4	Toronto, ON	552	20	1	1400	625	25,159	1	100	12.3	0.0	100
2019	5	Hamilton, ON	837	20	3	1200	930	107,577	803	99.3	56.5	0.0	100
2019	6	Oakville, ON	518	20	3	1000	477	22,117	0	100	7.0	0.0	100
2022	7	Hamilton, ON	403	50	2	1380	350	41,100	86	99.8	32.1	0.2	99.4
2022	8	Oshawa, ON	449	20	1	1200	550	7536	0	100	20.1	0.0	100
2022	9	Levis, QC	1809	20	3	3000	1143	2598	1	100	46.4	0.2	99.6
2022	10	Hamilton, ON	679	40	2	1380	463	19,447	73	99.6	168.7	1.0	99.3
2022	11	Sorel-Tracy, QC	725	20	9	1000	506	9527	0	100	89.3	0.0	100

### Abundance of organisms

**Fig. 2:**
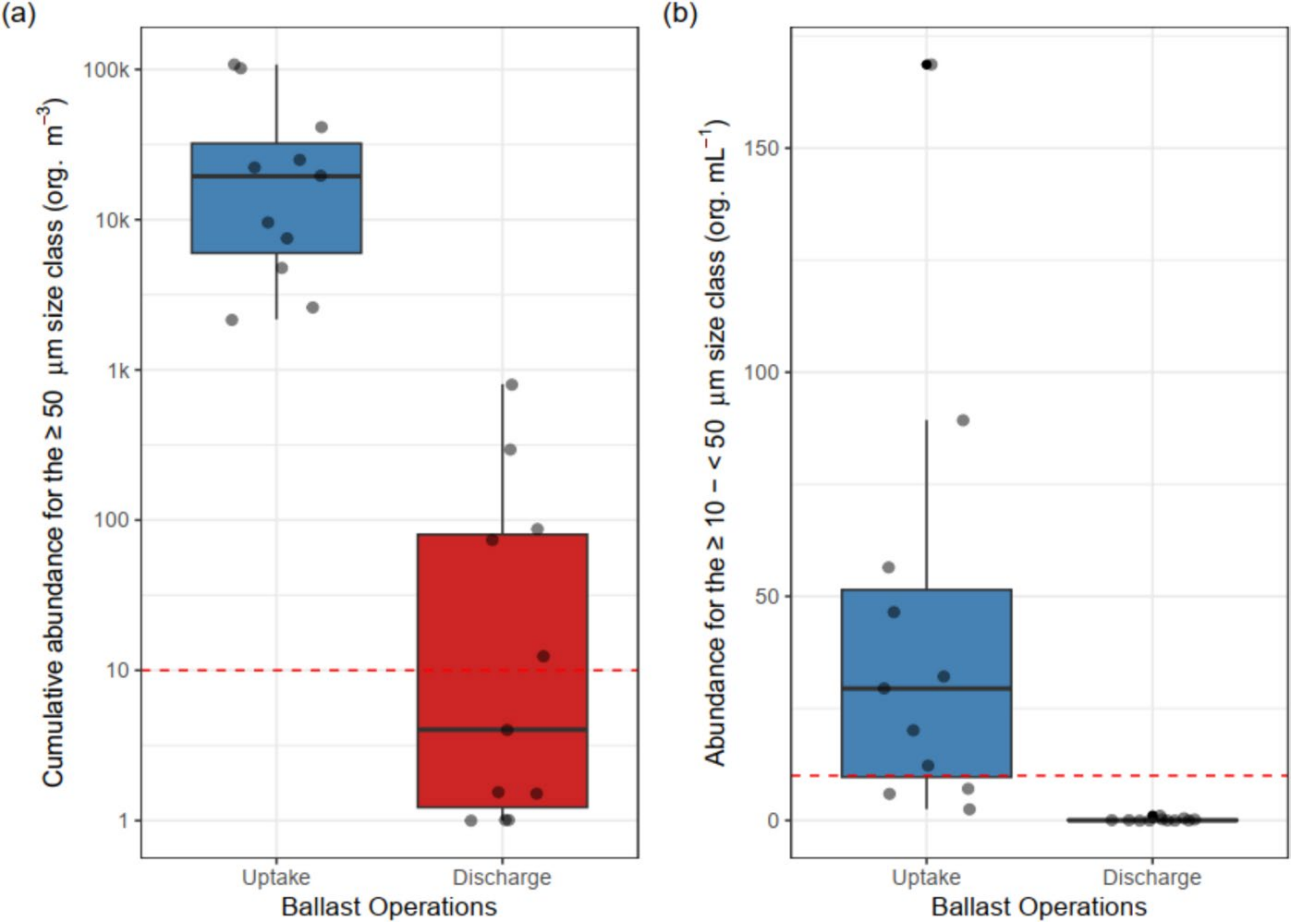
Box plots showing the median organism abundances (black line) for the two size classes in ballast water samples from ships transiting the Great Lakes-St. Lawrence River region, 5 th and 95 th percentiles (whiskers), and outliers for the large size class (≥ 50 μm) (**a**) and the small size class (≥ 10 to < 50 μm) (**b**). Note the y-axis for the large size class (**a**) is in log scale for better visualization. The dashed lines represent the limits set by Regulation D-2

Our assessment of BWMS performance on ships operating in the GLSLR region revealed BWMS regularly achieve significant reductions (> 98%) in organism concentrations, although the discharge samples did not consistently meet Regulation D-2. For the large-size class, uptake samples (untreated ballast water, *n* = 11) ranged from 2168 organisms/m^3^ to 107,577 organism/m^3^ with a median of 19,447 organisms/m^3^ (Fig. 2). Six of the corresponding treated discharge samples were below the limit of Regulation D-2, one was close to the limit (12 organisms/m^3^, with confidence intervals spanning the limit), and four were above the limit (73–803 organisms/m^3^; Fig. 2).

While Regulation D-2 sets a discharge limit of less than 10 viable organisms per cubic meter for the large size class, evaluating BWMS performance only based on discharge samples may not fully capture the extent of organism removal. Calculating the percentage reduction in organism abundance provides a standardized assessment of treatment efficacy that accounts for initial variation in uptake water. Furthermore, by reporting percentage reduction, we highlight the pronounced treatment effect of BWMS even when Regulation D-2 is not met.

In this study, failure to meet Regulation D-2 occurred exclusively when ballast was loaded at Hamilton Harbor—one of the most eutrophic ports in the Great Lakes (Bowen & Currie, 2017) — highlighting that local environmental conditions may affect BWMS effectiveness. Overall, discharge samples for this size class had > 99.3% reduction compared to their paired untreated uptake samples (Table 1). The observed 36% failure rate with the D-2 limit for the large-size class aligns with recent studies (Bailey et al., 2022; Outinen et al., 2024), where failure rates were observed for 50 and 30% of ballast water discharge samples following use of BWMS. This poor rate of compliance is offset by the pronounced reduction in organism abundances (> 99% for large-size class, > 98% for small-size class) in treated discharge samples compared to their paired, untreated uptake sample demonstrating high efficacy of BWMS (Table 1).

For the small-size class, uptake samples (untreated ballast water, *n* = 11) ranged from 3.0 to 168.7 organisms/mL with a median of 29 organisms/mL — noting that on uptake, one sample was below the limit of Regulation D-2, while three were close to the limit (7–12 organisms/mL, with confidence intervals spanning the limit) and seven were above the limit (20–169 organisms/mL) (Fig. 2). All treated discharge samples (*n* = 11) were below the limit of Regulation D-2 and had > 98% reduction in small size class organism abundances compared to their paired untreated uptake samples (Table 1). The high rate of compliance for the small size class (100%) is similar to results reported in previous studies (< 2% failure rate) (Casas-Monroy & Bailey, 2021; Drillet et al., 2023; Outinen et al., 2024).

The differences in compliance rates for the large and small size classes can be attributed to both biological and regulatory factors. First, small-size-class organisms such as protists and phytoplankton typically have delicate cellular structures that may be easily compromised during the mechanical filtration phase of BWMS treatment (Molina et al., 2018) while large-size-class organisms such as copepods and other microcrustaceans can have more flexible exoskeletons, particularly after molting, which may allow individuals to pass through filters unharmed (Briski et al. 2014). Some studies have also shown that, in general, UV irradiation is more effective against smaller unicellular organisms due to their higher surface-area-to-volume ratio, reducing their ability to develop protective mechanisms, and the shorter path length for UV photons to reach critical cellular targets. These factors may contribute to a stronger killing effect of UV irradiation on smaller organisms compared to larger ones (Hader 2000; Wang et al. 2025). Second, the relative stringency of the D-2 limits plays a notable role: while uptake abundances for the small size class were no more than 20 × the discharge limit, uptake abundances for the large size class were up to 10,000 × greater than the discharge limit, indicating that Regulation D-2 imposes more demanding requirements for the large size class while compliance is more achievable for the small size class (some uptake samples met the limit before any treatment was applied). Future research could explore whether adjusting discharge limits for smaller organisms could lead to a more balanced regulatory framework. However, determining an optimal threshold was beyond the scope of this study.

### Generalized linear models

Comparison of the negative binomial and Quasi-Poisson distribution models showed that the negative binomial models did not provide an acceptable fit for either the large- or small-size-class data while the Quasi-Poisson models showed a satisfactory fit for both response variables as shown in figures (Online Resource 1 and 2). The residual deviance of the Quasi-Poisson models was also much lower than that of the negative binomial models, suggesting that the Quasi-Poisson model explains a statistically- significant amount of variation in the two response variables as shown in tables (Online Resources 1 and 2).

For the large size class, uptake abundance (organisms/m^3^) and mesh size (µm) were strong predictors of zooplankton abundance in discharge samples (positive relationship, *p* value < 0.05) (Table 2). Factors such as ballast age (days), disinfection type, and BWMS age (years) were inversely related to discharge abundance, although the relationship was statistically significant only for ballast age (*p* value < 0.05; Table 2).

**Table 2:** Parameter estimates from the quasi-Poisson generalized linear model for discharge abundance of organisms in the large size class (≥ 50 µm)

Coefficients	Estimate	SE	*t* value	Pr(>|t|)
Intercept	3.129e + 01	6.482e + 01	0.48	0.71
Disinfection type	− 2.651e + 01	2.340e + 00	− 11.33	0.06
Ballast age (days)	− 6.798e + 00	4.951e − 01	− 13.73	0.04 *
BWMS age	− 1.131e + 01	9.049e − 01	− 12.5	0.05
Sampling duration	2.795e + 01	5.668e + 00	4.93	0.13
EXO turbidity	6.615e − 01	1.354e − 01	4.89	0.13
EXO fDOM	− 2.332e + 00	9.307e − 01	− 2.51	0.24
UVT	− 5.897e − 01	6.489e − 01	− 0.91	0.53
Uptake Zoop. Abun	1.986e − 04	4.770e − 06	41.63	0.02 *
Mesh size	1.920e + 00	7.688e − 02	24.97	0.03 *

*SE* Standard Error, *Pr* probability. Level of statistical significance: * = *p* < 0.05, *EXO*  multiparameter sonde to measure abiotic parameters,  *fDOM*  fluorescent dissolve organic matter, *UVT*  ultraviolet transmittance

Regarding the small size class, the statistical analysis revealed that ballast age (days) was significantly, inversely related to the number of live cells/mL in ballast discharge samples (*p* value < 0.05; Table 3) The EXO fDOM exhibited a marginally positive relationship with small-size-class abundance, suggesting potential interactions between dissolved organic matter and plankton communities. However, this relationship may also be influenced by unmeasured factors. To our knowledge, this is the first research comparing samples before and after BWMS treatment identifying the abundance of organisms in uptake water as a key determinant of discharge abundance (and therefore, compliance outcome), while the importance of mesh size is consistent with previous studies of ballast water discharge (without paired uptake samples)(Casas-Monroy & Bailey, 2021; Outinen et al., 2024). Furthermore, factors such as ballast age (days) were also consistent with previous studies examining ballast water exchange (Burkholder et al., 2007; Cordell et al., 2008).

**Table 3:** Parameter estimates from the quasi-Poisson generalized linear model for discharge abundance of organisms in the small size class (≥ 10 to < 50 µm)

Coefficients	Estimate	SE	t value	Pr(>|t|)
Intercept	− 211.39	99.80	− 2.12	0.17
Disinfection type	− 7.90	6.89	− 1.15	0.37
Ballast age (days)	− 1.84	0.32	− 5.68	0.03*
BWMS age	1.55	1.08	1.43	0.29
Sampling duration	10.28	3.92	2.62	0.12
EXO turbidity	0.084	0.06	1.37	0.31
EXO fDOM	6.45	1.69	3.83	0.06
UVT	1.91	1.01	1.88	0.20
Uptake phytoplankton abundance	− 0.02	0.03	− 0.62	0.60

*SE* standard error, *Pr* probability. Levels of statistical significance: *p < 0.05, *EXO *multiparameter sonde to measure abiotic parameters,  *fDOM* fluorescent dissolve organic matter, *UVT *ultraviolet transmittance

### BWMS information

During sampling visits, different technical matters were observed in relation to BWMS operation and maintenance. On one ship, BWMS alarms were observed during uptake (TRO minor alarm) and discharge (sticky neutralizer valve). On a second ship, the control panel was overheating. Another ship crew had intentionally reduced the number of UV lamps during low-flow ballast water uptake and discharge to reduce power demand; however, with fluctuating ballast pumping rates, the flow rate at least occasionally exceeded the reduced treatment capacity of the BWMS. These observations indicate that improvements in compliance rates might be achieved through crew training and more accessible information on BWMS operation, troubleshooting, and maintenance.

### Diversity

For the large size class, 11 paired samples (22 individual samples) were analyzed using microscopy and metabarcoding sequencing. Microscopy identified living organisms in all 11 uptake samples and 8 discharge samples, with no living organisms observed in 3 discharge samples (Online Resource 3). Shannon diversity indices calculated from microscopic counts revealed significantly lower diversity of living organisms in treated discharge samples compared to uptake samples (two-sample Welch’s *t* tests, *p* < 0.05; *n* = 22), indicating reduced taxonomic richness and evenness. Metabarcoding sequencing generated genomic data from only five uptake and one discharge samples. Molecular analysis of preserved samples also showed higher diversity in uptake samples (mean Shannon index = 1.2, *n* = 5) compared to the single discharge sample (Shannon index = 0.8, *n* = 1).

For the small size class, 11 paired samples (22 individual samples) were analyzed using microscopy and metabarcoding sequencing (Online Resource 4). Microscopy found intact organisms in all preserved uptake and discharge samples, despite the absence of living cell observations in discharge samples at the time of collection. Shannon diversity indices calculated from preserved microscopic counts revealed marginally higher diversity in discharge samples than uptake samples, though this difference was not statistically significant (two-sample Welch’s *t* tests, *p* > 0.05; *n* = 22) (Table 4). Metabarcoding sequencing generated genomic data from five uptake and the five corresponding paired discharge samples. Molecular analysis revealed minimal change in diversity between uptake and discharge samples (*p* > 0.05; *n* = 10). Overall, the Shannon diversity indices indicate that the ballast water treatment and discharge process significantly reduce diversity in the large size class, while a negligible effect was observed for the small size class (Table 4).

**Table 4:** Comparison of Shannon diversity indices between uptake and discharge ballast water samples across different taxonomic groups for both organism size classes using Welch’s *t* test; *a* and *b* microscopy, *c* and *d*, molecular analysis, noting that *a* is based on assessment of live organisms while the rest were assessments of preserved samples

Group	Mean uptake	Mean discharge	*t* value	DF	*p* value
*a* ≥ 50 μm size class	1.2	0.5	− 3.8	15	0.001
*b* ≥ 10 to < 50 µm size class	1.6	1.8	0.53	18.1	0.6
*c* ≥ 50 μm size class	1.2	0.8	-	-	-
*d* ≥ 10 to < 50 µm size class	1.3	1.2	− 0.29	11.0	0.8

The complementary application of microscopy and molecular techniques revealed important methodological challenges and advantages for ballast water research. One of the most evident limitations was the DNA recovery, with only 50% success rate in uptake samples with abundances > 1000 organisms per m^3^ indicating potential problems with the sample collection and analysis workflow rather than treatment effects. The poor DNA recovery from discharge samples may be due to multiple factors such as the low organism abundances post-treatment, DNA degradation from BWMS treatments (e.g., chlorine or UV irradiation), or losses during sample processing. Comparison between microscopy and molecular analysis revealed that molecular methods detected a greater number of taxonomic groups and genera in uptake samples, although this likely includes DNA from both live and dead organisms. Conversely, traditional microscopy can provide counts of living organisms through direct observation, but the volume of sample that can be assessed is limited.

Across all samples, microscopy identified six taxonomic groups (large size class) and 56 genera (small size class) in discharge samples, while molecular analysis detected 8 taxonomic groups and 73 unique genera, respectively. The two methods overlapped in identifying four taxonomic groups of the large size class and 24 genera of the small size class in uptake samples and four taxonomic groups and genera of each size class in discharge samples. For the large size class, microscopy found 25% fewer taxonomic groups in discharge samples compared to uptake; while molecular analysis found 74% fewer, the results are confounded by poor DNA recovery (1 discharge sample vs. 5 uptake samples). For the small size class, microscopy showed a 4% increase, while molecular analysis revealed an 84% decrease, in genera in discharge samples compared to uptake (Online Resource 5).

These findings highlight the complementary strengths of microscopy and molecular techniques in assessing treatment efficacy and biodiversity changes, respectively, with molecular methods potentially offering a more sensitive approach for detecting and identifying smaller or juvenile organisms. While metabarcoding shows promise for ballast water analysis, its limitations suggest that it should complement, rather than replace, traditional methods (Egan et al., 2015; James et al., 2024). Combining molecular and microscopic approaches provides the most comprehensive characterization of ballast water biodiversity, for better understanding of treatment efficacy and improving strategies to mitigate the risk of non-native species introductions in ballast water discharge.

## Conclusions

Examining paired uptake and discharge samples revealed a pronounced treatment effect of ballast water management systems (BWMS) installed on ships transiting the Great Lakes-St. Lawrence River (GLSLR), even when Regulation D-2 was not met. The results demonstrate that BWMS consistently reduced the abundance of live organisms by > 98% for both regulated size classes, substantially lowering the risk of introducing and spreading harmful aquatic species. All discharge samples met the Regulation D-2 limit for the small size class (≥ 10 to < 50 μm), while four samples (36%) exceeded the limit for the large size class (≥ 50 μm). Uptake abundance and mesh size were identified as key predictors of discharge abundance, particularly for the large size class. With all four tests with exceedances being sourced from Hamilton Harbour, Lake Ontario, this research indicates that highly productive, eutrophic waters may pose a challenge for achieving compliance with Regulation D-2. This study also revealed significant effects of BWMS on plankton community diversity, with lower taxonomic richness and evenness in discharge samples for the large size class and a decrease in genera for the small size class compared to uptake. This study highlights the complementary strengths of microscopy and molecular techniques in assessing treatment efficacy and biodiversity changes, respectively, and the importance of considering both uptake and discharge samples in evaluating ballast water treatment efficacy (also reinforced by the BWMS Code, which requires comparative analysis during certification testing to validate system performance under operational conditions (IMO, 2018)). Future research should assess water quality of ballast source environments in relation to BWMS efficacy and type approval testing requirements with a view to improve technology and could explore whether adjusting discharge limits for smaller organisms could lead to a more balanced regulatory framework.

## Supplementary Information

Below is the link to the electronic supplementary material.

Supplementary file1 (PDF 366 KB)

## Data Availability

Data can be available anytime upon request.
