# Supplementary material for: Effectiveness of ballast water management systems in the Great Lakes based on a paired uptake-discharge sample design
Source: Environ Monit Assess. 2025 May 2;197(6):618. doi: 10.1007/s10661-025-14032-3 (PMC12048448; doi:10.1007/s10661-025-14032-3)
Supplement: Supplementary file 1 — Supplementary file1 (PDF 366 KB) [file 10661_2025_14032_MOESM1_ESM.pdf]

Effectiveness of ballast water management systems in the Great Lakes based on a paired uptake-discharge sample design

Oscar Casas-Monroy[1*], Jiban C. Deb[1,2], Jocelyn Kydd[1], Robin Rozon[1], Sean Yardley[1], Sophie Crevecoeur[3], Sarah A. Brown[4], John A. Darling[5], and Sarah A. Bailey[1]

[1]Great Lakes Laboratory for Fisheries and Aquatic Sciences, Fisheries and Oceans Canada, 867 Lakeshore Road, Burlington, ON, L7S 1A1, Canada

[2]Northern Hardwoods Research Institute Inc. 165 Boulevard Hébert, Edmundston, NB, E3V 2S8, Canada

[3]Watershed Hydrology and Ecology Research Division, Environment and Climate Change Canada, 867 Lakeshore Road, Burlington, ON, L7S 1A1, Canada

[4]Oak Ridge Institute for Science and Education, P.O. Box 117, Oak Ridge, TN 37831, USA

[5]USEPA Office of Research and Development, Center for Environmental Measurement and Modeling, 109 T.W. Alexander Dr, RTP, NC 27709

Correspondence: *Oscar Casas-Monroy

Supplementary Table 1 Supporting Information: Goodness-of-fit values of Quasi-Poisson models with nearly zero residual deviance for organisms in the size classes ≥ 50 µm (typically comprised of zooplankton) and ≥ 10 to < 50 µm (typically comprised of phytoplankton)

Models	Null Deviance	Residual Deviance
Zooplankton	3.46E+03	4.80E-10
Phytoplankton	5.07E+00	4.92E-10

Supplementary Table 2 Supporting Information: Goodness-of-fit values of Negative Binomial models with larger residual deviance for organisms in the size classes ≥ 50 µm (typically comprised of zooplankton) and ≥ 10 to < 50 µm (typically comprised of phytoplankton)

Models	Null Deviance	Residual Deviance
Zooplankton	3.46E+03	1.36E+00
Phytoplankton	6.97E+00	1.90E+00

Supplementary Table 3 Supporting Information: List of taxonomic groups of organisms in the ≥ 50 µm size class identified in each ballast operation using bright light microscopy and DNA metabarcoding

	Microscopy		Molecular analysis	
**Taxon**	**Uptake (n=11)**	**Discharge (n=8)**	**Uptake (n=5)**	**Discharge (n=1)**
Annelida	x		x	
Anthozoa			x	
Arachnida	x	x	x	x
Bivalvia	x	x	x	
Blattodea			x	
Chironomidae	x		x	x
Ciliophora	x			
Cirripedia	x			
Coleoptera			x	x
Copepod	x	x	x	x
Copepod nauplii	x	x		
Culicidae			x	
Diplostraca	x	x	x	x
Diptera			x	
Glomerida			x	
Hemiptera			x	
Hydrozoa			x	
Hymenoptera			x	
Insecta			x	x
Isopoda			x	
Lepidoptera			x	
Malacostraca			x	
Mycetophilidae			x	
Nematoda	x		x	
Ostracoda	x		x	
Platyhelminthes			x	
Psychodidae			x	
Rotifera	x	x	x	x
Sciaridae			x	
Scyphozoa			x	
Sphaeroceridae			x	
Tartigrada			x	
Thysanoptera			x	
Unknown	x		x	x
Number of taxonomic groups	13	6	31	8

* Note these organisms are not excluded by the metabarcoding analysis itself but that the method does not distinguish between life stages.

Supplementary Table 4 Supporting Information: List of genera of organisms in the ≥ 10 to < 50 µm size class identified in each ballast operation using bright light microscopy and DNA metabarcoding; n = number of samples analyzed with each technique or preservation method

Context: To address challenges associated with keeping filters frozen, we tested alternative preservation methods for DNA analysis of ballast water samples from uptake and discharge for this size class. Filters were preserved using three methods dry preservation, ethanol, and RNA later. Filters stored in ethanol and RNA later were frozen at -80°C until further processing, whereas the dry preservation method required no freezing. Statistical analyses showed no significant differences in the number of reads per species or genera among the three preservation methods. Therefore, only the results from the dry preservation method are presented in this study.

	**Microscocopy**		**Molecular analysis**	
	**Uptake (n=11)**	**Discharge (n=11)**	**Uptake (n11)**	**Discharge (n=11)**
**Genera**	**Lugol's solution**	**Lugol's solution**	**Dried (n=5)**	**Dried (n=2)**
Acanthamoeba			x	
Achnanthes	x	x		
Achnanthidium			x	
Acineta				
Actinastrum	x			
Actinocyclus				
Actinotaenium			x	
Acutodesmus				
Alexandrium			x	
Amoebophrya			x	
Amphora	x	x	x	
Amylax		x		
Anabaena	x	x		
Ankistrodesmus			x	
Anteholosticha				
Arcuospathidium				
Asterionella		x	x	
Asterionellopsis		x		
Atractomorpha				
Aulacoseira	x	x	x	
Bacillaria	x		x	
Biecheleria				
Bistichella				
Botryococcus			x	
Bracteacoccus				
Brooklynella				x
Carchesium			x	
Carteria	x		x	
Ceratiomyxella			x	
Ceratium	x	x	x	x
Cercomonas				

Chaetoceros				
Chaetopeltis				
Chlamydomonas		x	x	
Chlorella	x	x		
Chlorochytrium			x	
Chloroidium				
Chloromonas				
Choanoflagellates				
Choricystis			x	
Chromulina				
Chroococcus	x			
Chroomonas				
Chrysochromulina	x	x		
Closterium		x	x	
Coccolithus			x	
Cocconeis	x	x	x	
Codonellopsis			x	
Coelastrum	x	x	x	
Coelosphaerium	x			
Colpodella				x
Colpodidium			x	
Condylostoma			x	
Coscinodiscus	x	x		
Cosmarium	x	x		
Cothurnia			x	
Cryptomonas	x	x	x	x
Cryptoperidiniopsis			x	
Cyclotella	x	x	x	
Cylindrotheca		x	x	
Cymatopleura			x	
Cymbella	x	x	x	
Desmodesmus			x	
Diaphanoeca			x	
Diatoma	x	x	x	
Dictyocha		x		
Dictyochloropsis			x	
Didinium			x	
Dinobryon	x			
Diplopsalis	x			
Discostella			x	
Durinskia				
Eimeria				
Emiliania			x	

Encyonema			x	
Epistylis				
Euglenoid	x	x		
Flamella			x	x
Fragilaria			x	
Fragilariopsis			x	x
Franceia				
Glenodiniopsis				
Glenodinium	x	x		
Gloeocapsa	x	x		
Golenkinia				
Gomphonema	x	x	x	
Gonatozygon			x	
Goniomonas				
Gonium				
Gonyostomum				
Goussia				
Gymnodinium	x	x	x	
Gymnophrys			x	
Gyrosigma	x	x	x	
Haematococcus	x	x		
Halteria				
Hemiophrys			x	
Heterocapsa			x	
Hydrodictyon			x	
Hydrurus				
Jadwigia			x	
Karlodinium			x	x
Katablepharis		x		
Lembadion			x	
Lepocinclis	x			
Leptocylindrus		x		
Leucocryptos			x	
Lobochlamys				
Lophodinium	x			
Loxophyllum				
Mallomonas	x		x	
Massisteria				
Melosira	x	x		
Merismopedia		x		
Mesotaenium			x	
Micractinium				
Micrasterias			x	x

Monodus				
Monoraphidium			x	
Mougeotia		x	x	
Mychonastes			x	
Naegleria			x	
Nannochloropsis			x	
Navicula	x	x	x	
Neidium			x	
Neoceratium			x	
Neochlorosarcina				
Neoperezia			x	
Nitzschia	x	x	x	
Nuclearia			x	
Ochromonas			x	
Oocystella				
Oocystis	x	x	x	x
Ophryocystis				
Oscillatoria	x	x		
Paralia		x		
Paraphysomonas			x	x
Pectodictyon				
Pediastrum	x	x		
Pedinomonas				
Pelagodinium			x	
Pelagostrobilidium			x	
Penium				
Peridiniopsis	x			
Peridinium	x	x	x	
Pfiesteria			x	x
Phacotus			x	
Phacus	x	x		
Phaeocystis			x	
Phialina				
Pinnularia	x	x		
Placoneis			x	
Planothidium			x	
Plasmodiophora			x	
Polyedriopsis				
Protodesmus				
Protoperidinium			x	
Protosiphon				
Pseudocharaciopsis				
Pseudocohnilembus				

Pseudonitzschia				
Pseudo-nitzschia		x		
Pseudopirsonia			x	x
Pseudourostyla				
Radiococcus			x	
Reticulosphaera				
Rhizidiomyces				
Rhodomonas	x	x		
Rhogostoma			x	
Rigidothrix			x	
Rimostrombidium			x	
Salpingoeca			x	
Sarcinochrysis				
Scenedesmus	x	x	x	x
Scrippsiella			x	x
Sellaphora			x	
Skeletonema			x	
Spathidium			x	
Spermatozopsis			x	
Sphaerocystis	x			
Spirogyra	x	x		
Spumella			x	
Staurastrum	x	x	x	
Stauroneis				
Staurosira			x	
Stephanodiscus	x	x	x	
Stichococcus			x	x
Stigeoclonium			x	
Stokesia				
Strombidium			x	
Surirella	x	x		
Symbiodinium			x	x
Synedra	x	x	x	
Synura			x	
Tabellaria	x	x		
Teleaulax			x	
Telotrochidium			x	
Tetracystis			x	
Tetraedron		x		
Tetraselmis				
Tetrastrum		x		
Thalassiosira			x	
Tiarina			x	

Tintinnopsis	x	x	x	
Trachelomonas	x			
Trachydiscus				
Trebouxia			x	
Trochiscia	x	x		
Ulkenia				
Unknown			x	x
Uroglena			x	
Uroleptus				
Uronema			x	
Vermamoeba			x	
Volvox			x	
Vorticella			x	x
Wilbertia			x	
Wislouchiella				
Woloszynskia			x	
Zoothamnium			x	x
Number of genera	54	56	120	19

Supplementary Table 5 Supporting Information: summary of percentage of removed taxonomic groups in the ≥ 50 µm size class and genera ≥ 10 to < 50 µm size class, in the identified in each ballast operation using bright light microscopy and DNA metabarcoding

	≥50 µm size class			10 to 50 µm size class		
**Technique**	**Uptake**	**Discharge**	**Percentage of removed organisms**	**Uptake**	**Discharge**	**Percentage of removed organisms**
**microscopy**	8	6	25	54	56	-3.7
**molecular analysis**	31	8	74.2	120	19	84.2

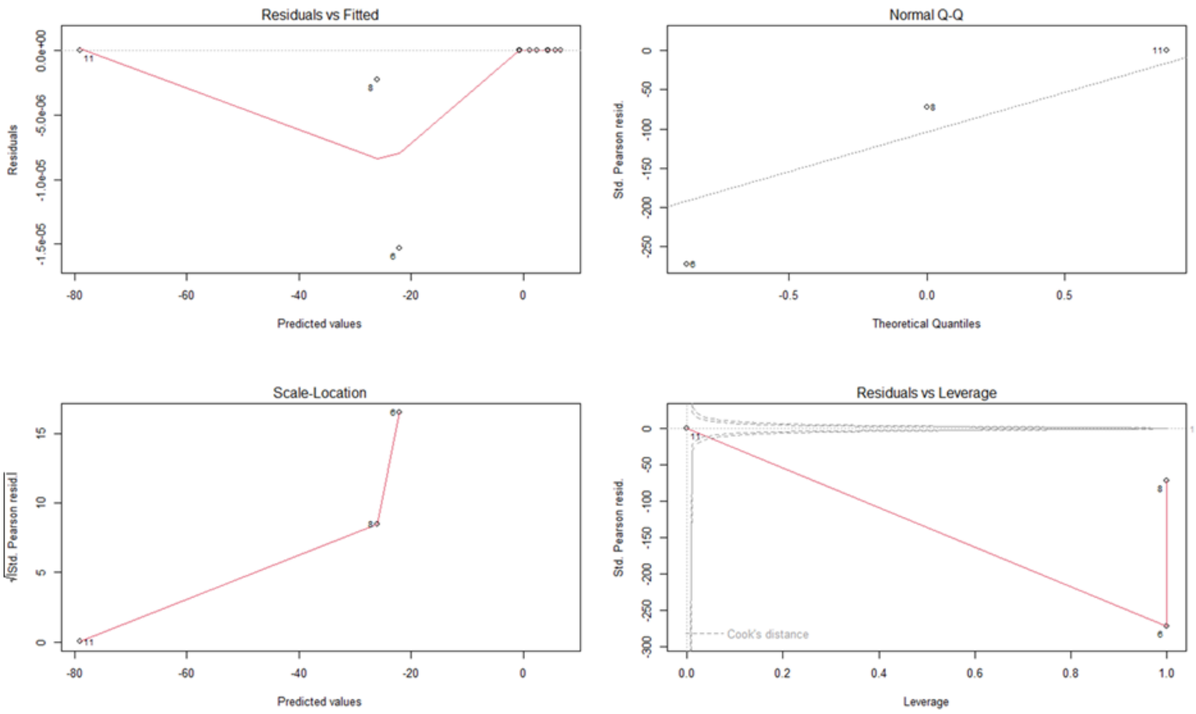

Supplementary Figure 1. Diagnostic plots of Quasi-Poisson model for organisms in the ≥ 50 μm size class (typically comprised of zooplankton)

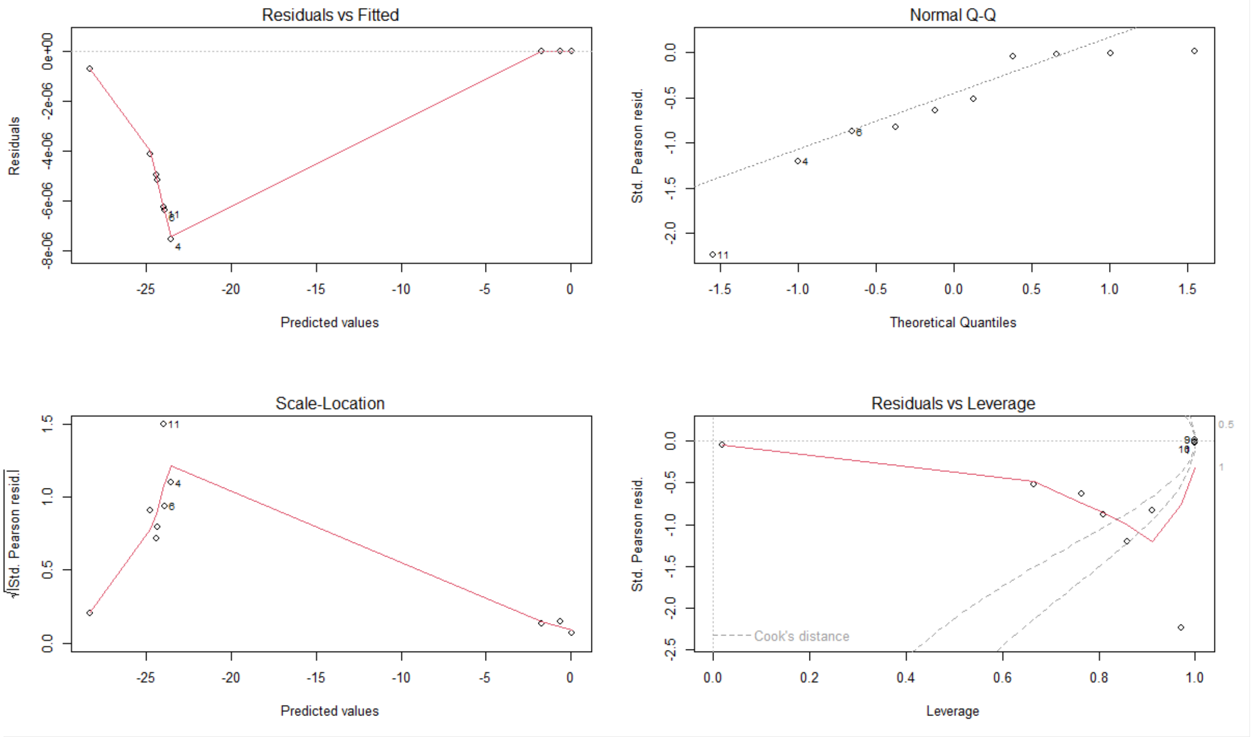

Supplementary Figure 2. Diagnostic plots of Quasi-Poisson model for organisms in the ≥ 10 to < 50 μm size class (typically comprised of phytoplankton).
